# Optical conductivity-based ultrasensitive mid-infrared biosensing on a hybrid metasurface

**DOI:** 10.1038/s41377-018-0066-1

**Published:** 2018-09-26

**Authors:** Yibo Zhu, Zhaoyi Li, Zhuang Hao, Christopher DiMarco, Panita Maturavongsadit, Yufeng Hao, Ming Lu, Aaron Stein, Qian Wang, James Hone, Nanfang Yu, Qiao Lin

**Affiliations:** 1 Department of Mechanical Engineering, Columbia University, New York, NY 10027 USA; 2 Department of Applied Physics and Applied Math, Columbia University, New York, NY 10027 USA; 3 Department of Chemistry and Biochemistry, University of South Carolina, Columbia, SC 29208 USA; 4 National Laboratory of Solid State Microstructures, College of Engineering and Applied Sciences, and Collaborative Innovation Center of Advanced Microstructures, Nanjing University, Nanjing, 210093 China; 5 Center for Functional Nanomaterials, Brookhaven National Laboratory, Upton, NY 11973 USA

## Abstract

Optical devices are highly attractive for biosensing as they can not only enable quantitative measurements of analytes but also provide information on molecular structures. Unfortunately, typical refractive index-based optical sensors do not have sufficient sensitivity to probe the binding of low-molecular-weight analytes. Non-optical devices such as field-effect transistors can be more sensitive but do not offer some of the significant features of optical devices, particularly molecular fingerprinting. We present optical conductivity-based mid-infrared (mid-IR) biosensors that allow for sensitive and quantitative measurements of low-molecular-weight analytes as well as the enhancement of spectral fingerprints. The sensors employ a hybrid metasurface consisting of monolayer graphene and metallic nano-antennas and combine individual advantages of plasmonic, electronic and spectroscopic approaches. First, the hybrid metasurface sensors can optically detect target molecule-induced carrier doping to graphene, allowing highly sensitive detection of low-molecular-weight analytes despite their small sizes. Second, the resonance shifts caused by changes in graphene optical conductivity is a well-defined function of graphene carrier density, thereby allowing for quantification of the binding of molecules. Third, the sensor performance is highly stable and consistent thanks to its insensitivity to graphene carrier mobility degradation. Finally, the sensors can also act as substrates for surface-enhanced infrared spectroscopy. We demonstrated the measurement of monolayers of sub-nanometer-sized molecules or particles and affinity binding-based quantitative detection of glucose down to 200 pM (36 pg/mL). We also demonstrated enhanced fingerprinting of minute quantities of glucose and polymer molecules.

## Metasurfaces: mid-infrared biosensors

A highly sensitive glucose sensor has been constructed from a functionalized graphene-metallic hybrid metasurface. Pioneered by a US-Chinese collaboration from Columbia University, University of South Carolina, Brookhaven National Laboratory and Nanjing University the device by detecting the changes in graphene optical conductivity, enables ultrasensitive detection of glucose concentrations as small as 200 pM via a small shift in its plasmonic resonant wavelength which is then measured. Importantly, the new sensing principle overcomes the detection limit defined by the molecular weight or the changes in local refractive index, which for a long time has impeded the development of more sensitive optical biosensors. The device consists of a monolayer of graphene covering an array of gold nanorods, atop a platinum-silicon dioxide-platinum sandwich that serves as an optical cavity. When the graphene is functionalized with boronic acid which serves as a glucose binding agent, the device’s wavelength response was seen to clearly red shift with increasingly glucose concentration. Experiments indicate a dynamic range of measurement of over 6 orders of magnitude from 2nM to 10mM.

## Introduction

Optical transducers are one of the most promising candidates for label-free, sensitive, and specific on-chip biosensing.^1–3^ Optical devices are unique in their ability to not only detect binding between biochemical molecules but also probe their molecular structures^4–8^. Most optical sensors, such as those based on surface plasmon resonance^9^ or optical fibers^10^, rely on the detection of changes in refractive indices to quantify analytes or monitor binding processes, but they unfortunately lack the sensitivity to probe the binding involving low-molecular-weight molecules^11,12^. Spectroscopic methods can reveal molecular fingerprints and quantify analytes according to signal intensities but are limited by noisy background and low sensitivity as well^13,14^. Non-optical devices such as field effect transistors may provide more sensitive alternatives^11,15–17^ at the expense of molecular spectroscopic information, convenient wireless communication between optical devices, and other features of optical methods. Also, use of these devices may face other complexities in biosensing, such as unstable contact, electrochemical corrosion, current leakage, and reduced sensitivity in high-ionic-strength media^18–21^.

Very recently, with highly tunable electrical and optical properties, two-dimensional materials have been employed to construct novel optoelectronic devices^22–25^. In particular, graphene is very well-suited to develop optical modulators or photodetectors operating in the mid-infrared (mid-IR)^26–28^ range, where spectral fingerprints of many molecular bonds can be found. However, the potential of these new device architectures in biosensing has not been widely explored, especially for the detection of low-molecular-weight analytes. While many studies have reported enhancement of the mid-IR fingerprints of protein or polymer layers using metal or graphene plasmons^29–32^, to date very limited advances have been made in the measurement of the binding of low-molecular-weight analytes in the mid-IR range; such measurements are challenging because of the significant mismatch in size between their sub-nanometer size and micrometer-scale IR wavelengths^30,33^.

In this paper, we demonstrate optical conductivity-based mid-IR biosensing that allows the sensitive measurement of low-molecular-weight analytes as well as the enhancement of their spectral fingerprints. The sensors used in this work employ a hybrid metasurface consisting of monolayer graphene and a metallic nano-antenna array. They offer a unique set of advantages that are not simultaneously available from existing electrically or optically based devices. First, the plasmonic resonance frequency of the sensors varies with the carrier doping introduced by target molecules adsorbed on graphene; therefore, the sensor is not limited by the molecular mass and can sensitively detect low-molecular-weight molecules thanks to the high sensitivity of graphene to molecular doping^34,35^ Meanwhile, the optical measurements are readily amenable to wireless deployment and avoid the complexities involved in electrical measurements that are typically used to measure carrier doping. Second, the sensors can resolve molecules with similar molecular masses by differentiating the resonance shifts caused by the changes in graphene optical conductivity, which is not possible with refractive index-based sensors. Third, the hybrid metasurface features a quality factor that is insensitive to variation in graphene carrier mobility and thus can be more stable than the devices using graphene as their sole functional material, which in general are affected by the degradation of carrier mobility^36,37^. Finally, the sensors can act as substrates for surface-enhanced IR absorption (SEIRA) spectroscopy to enhance the fingerprints in the mid-IR range and facilitate the identification of analytes.

We demonstrated the use of these hybrid metasurface sensors for sensitive detection of low-molecular weight analytes. Sub-monolayers of sub-nanometer analytes were detected and differentiated from each other. Glucose from 2 nM to 20 mM was quantified via its reversible binding with boronic acid anchored on graphene. We experimentally and theoretically studied the relationship between the metasurface geometry, electric-field confinement, and sensor sensitivity. This led us to further optimize the sensor design and achieve a detection limit of 200 pM glucose by confining the electric-field on ultra-narrow (10 nm) suspended graphene. Additionally, we demonstrated enhanced fingerprinting of minute quantities of glucose and polymer molecules on the metasurface, which are otherwise unrecognizable without enhancement. This work opens a path to the development of high performance, multifunctional sensors using 2D–3D hybrid structures for biomedical applications.

## Results

### Sensor response to molecular doping

**Fig. 1:**
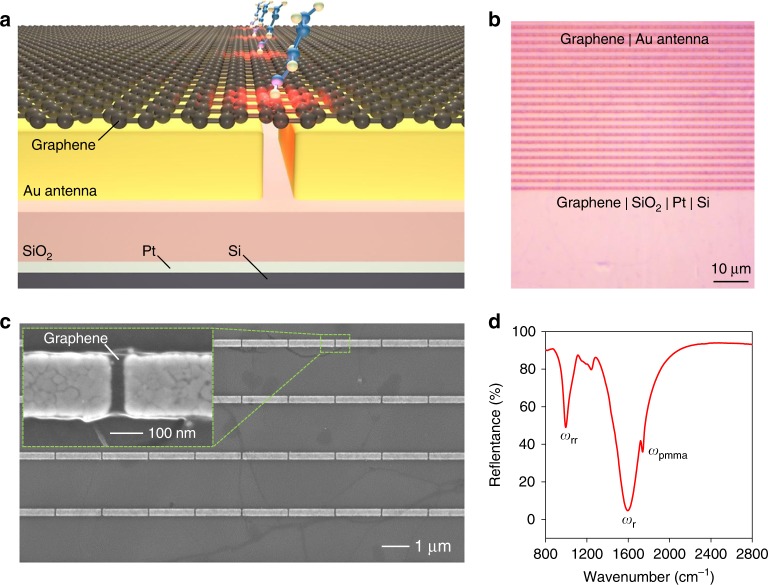
Architecture and optical properties of the hybrid metasurface. **a** Schematic of the graphene-metallic metasurface, with small molecules adsorbed on the suspended graphene. **b** Optical micrograph of one device, showing that the graphene monolayer is continuous and uniform over a large device area. **c** Scanning electron microscopy (SEM) image of graphene-coated nanorod antennas. Inset: SEM image of one antenna gap with suspended graphene. **d** Reflectance spectrum of a device showing a primary plasmonic resonance (*ω*_r_) at ~1500 cm^−1^, a resonance dip (*ω*_rr_) at 1000 cm^−1^ and a PMMA absorption peak (*ω*_pmma_) near 1700 cm^−1^

The hybrid metasurface consisted of an Au nanorod antenna array (length *L* = 1.64 μm) covered by monolayer graphene, atop a Pt mirror with a SiO_2_ spacer layer in between (Figs. 1a, b). The size of the gaps separating adjacent nanorod antennas was 30 nm. The SiO_2_ layer with the Pt mirror formed an optical cavity. When incident light excites oscillating dipoles (i.e., plasmonic resonance) in the antenna, the optical cavity can introduce imaging dipoles at the same distance underneath the Pt mirror, oscillating in opposite directions. A pair of such dipoles are coupled via near-field interactions and can form a quadrupole to suppress radiation loss in the far field and improve the quality factor of the plasmonic resonance band^38^. Benefiting from its excellent mechanical strength and flexibility, graphene conformed to the antennas and was suspended at the 30 nm gaps over a large device area (Fig. 1c and Figure S1). Compared to the previously reported devices that loaded graphene underneath the metallic antennas^26,28,39^, our device structure grants easier access of molecules to the graphene in the nanogap and thus is more suitable for sensing applications. Originating from the coupling between the SiO_2_ phonon resonance and the metasurface plasmon resonance, two resonance dips were respectively observed near 1500 cm^−1^ (*ω*_r_) and 1000 cm^−1^ (*ω*_rr_) in the mid-IR spectral region where the optical conductivity of graphene is highly tunable^22,40^. In this work, the peak near 1500 cm^−1^ was used as the primary plasmonic resonance band for biosensing, as it showed stronger absorption (Fig. 1d), up to 90% at *ω*_r_ with a signal to noise ratio exceeding 1000. Such a high signal-to-noise ratio ensures that the absorption bands of other materials, for example, the poly(methyl methacrylate) (PMMA) residue from device fabrication, would not significantly influence the profile of the plasmonic resonance band.

**Fig. 2:**
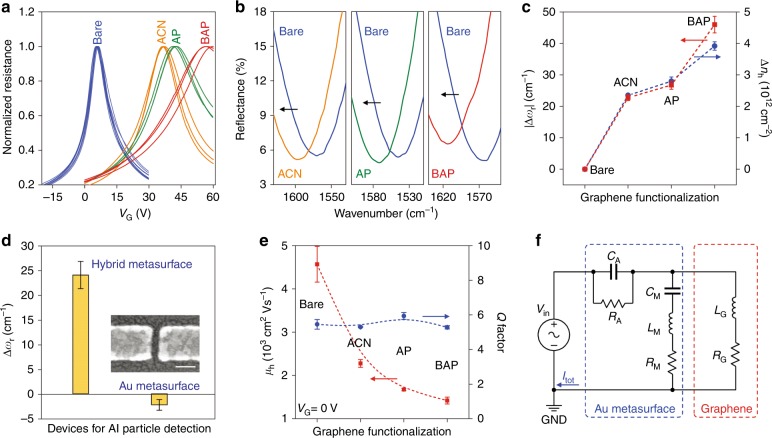
Detection of sub-nanometer molecules and metallic particles. **a** Graphene field effect transistor transport characteristics (resistance as a function of the gate voltage V_G_) and after exposure to different chemicals. **b** Metasurface reflectance spectra before and after chemical functionalization. **c** Dependence of the plasmonic resonance shifts on carrier densities of graphene. **d** Comparison of the resonance shifts caused by the deposition of 0.8 nm of Al on hybrid and bare Au metasurfaces. Inset: SEM image of one antenna gap with deposited Al nanoparticles. Scale bar: 100 nm. **e** Q-factors of the hybrid metasurface at different graphene carrier mobilities. **f** Equivalent circuit model of the hybrid metasurface. The symbols R, L, and C are, respectively, resistance, inductance, and capacitance; the subscripts A, M, and G represent air, metal antenna and graphene load, respectively. Error bars in (**c**)–(**e**) represent standard deviations achieved from nine measurements (three measurements on each of three independent devices)

To characterize the plasmonic resonance frequency of the hybrid metasurface as a function of the molecular doping of graphene, we first altered the carrier density of graphene by immobilizing small molecules on graphene. The hybrid metasurfaces with bare graphene were respectively exposed to the organic solvent acetonitrile (ACN) and two pyrene derivatives dissolved in ACN: amino-pyrene (AP, 217.27 g/mol) and boronic acid-pyrene (BAP, 246.07 g/mol). Raman spectroscopy and atomic force microscopy (AFM) indicated that the AP or BAP molecules were immobilized on graphene via π–π interactions (Figure S2a). Measurements of transport characteristics of graphene field effect transistors indicated that the pure ACN bath introduced p-doping to graphene, increasing the hole density (∆n_h_) by 2.3 × 10^12^ cm^−2^ (Fig. 2a). Immobilization of AP and BAP molecules produced additional increases in hole density of 0.46 × 10^12^ cm^−2^ and 1.58 × 10^12^ cm^−2^, respectively. Accordingly, *ω*_r_ shifted to higher wavenumbers by 23 cm^−1^, 27 cm^−1^, and 46 cm^−1^ after treatment with ACN, AP, and BAP, respectively (Fig. 2b). These blue shifts, opposite to the red-shifts found in typical plasmonic sensors, are attributed to the increases in the graphene optical conductivity caused by the molecular doping to graphene, corresponding to a more negative real part of the graphene permittivity^26^. The amount of the shift, |∆*ω*_r_|, as a function of the carrier density doped into graphene by the molecules (Fig. 2c), allowed us to quantify the binding of low-molecular-weight molecules, as demonstrated below.

To further examine the contributions of the changes in optical conductivity and refractive index to the shift of *ω*_r_, a sub-nanometer thick (0.8 nm) Al layer was vapor-phase deposited onto the bare Au metasurface (without graphene) and the hybrid metasurface, respectively. Different from the pyrene derivatives, the Al nanoparticles could be homogeneously coated on the bare sensor surface without graphene so that the effect of the refractive index changes on the shift of *ω*_r_ could be examined independently. As the work function of Al (4.28 eV) is lower than that of pristine graphene (4.6 eV), the Al nanoparticles upshifted the graphene Fermi level and donated electrons to graphene. The increase in carrier density caused by the Al nanoparticles was 2 × 10^12^ cm^−2^ (Figure S3b), leading to an apparent 24.1 cm^−1^ blue-shift of *ω*_r_ in the hybrid metasurface (Fig. 2d). After exclusion of the 2.2 cm^−1^ red-shift observed on the bare Au metasurface, which was due to the change of refractive index by the oxidized Al, the total blue-shift caused by the electron doping of the Al nanoparticles in the hybrid metasurface was 26.3 cm^−1^. In other words, the carrier doping-induced shift was ~12 times the shift induced by the change of the local refractive index. This confirmed the dominant contribution from graphene optical conductivity in the detection of these sub-nanometer subjects, especially for even smaller analytes, such as the pyrene derivatives (Figure S2b).

### Insensitivity of Q factor to graphene mobility

Besides the carrier density (*n*), the carrier mobility (*μ*) of graphene usually also influences the performance of graphene-based devices. For example, the intensity and quality factor of the plasmonic resonance excited on graphene nanoribbons vary significantly with graphene carrier mobility^31,37^. Interestingly, in our hybrid metasurface sensor, the profile of the plasmonic resonance peak did not show appreciable variations. In contrast, the carrier mobility of graphene decreased significantly after chemical treatment, as was evident from the considerably less sharp transfer characteristics (Fig. 2a). The quality factor (Q) of the plasmonic resonance peak remained approximately constant at 5.5 within a deviation of 5.6% (Fig. 2e), although the hole mobility was lowered by nearly 70%, from 4570 cm^2^ V^−1^s^−1^ to 1420 cm^2^ V^−1^s^−1^ at 0 V gate bias (see Supplementary Information section 1.2 for details). This is in agreement with the results of the finite-difference time-domain (FDTD) simulation (Figure S4). To better understand the effects of the carrier mobility, we also developed an equivalent circuit model of the hybrid metasurface (Fig. 2f), in which the current *I*_tot_ represents the reflectance of the metasurface. As expected, the frequency response of *I*_tot_ highly depended on the graphene carrier density but barely varied with the graphene carrier mobility (Figure S5c). Such weak dependence of *ω*_r_ on graphene carrier mobility can be explained by the insensitivity of graphene impedance to the carrier mobility. We note that the graphene impedance referred to here is not equivalent to the electrical resistance but is a function of graphene optical conductivity (see Section 3 in the Supplementary Information for details). The calculated impedance of the graphene load shows approximately 100 times stronger dependence on carrier density than on carrier mobility (Table S1). Therefore, the hybrid plasmonic sensor exhibits not only high sensitivity to adsorption of small molecules, but also excellent robustness against the degradation of the graphene carrier mobility.

### Reversible quantification of glucose

We then conducted measurements of glucose, a monosaccharide that serves as an important source of energy in cellular respiration, to demonstrate the sensitive quantification of small-molecule biomarkers using the hybrid metasurface. Recently, the affinity binding between glucose and boronic acid has attracted much attention in the development of next-generation, enzyme-free, and oxygen independent synthetic chemosensors^41^. However, affinity binding-based optical techniques for quantitative measurements of glucose at low concentrations have been scarce, possibly due to the low molecular mass and electrical neutrality of glucose. It has been shown that, upon glucose binding to boronic acid, the BAP could be converted from an electron-withdrawing group to an electron-donating group^42–45^. This transition of boronic acid is expected to give rise to changes in the graphene optical conductivity and allows for glucose sensing in the hybrid metasurface.

**Fig. 3:**
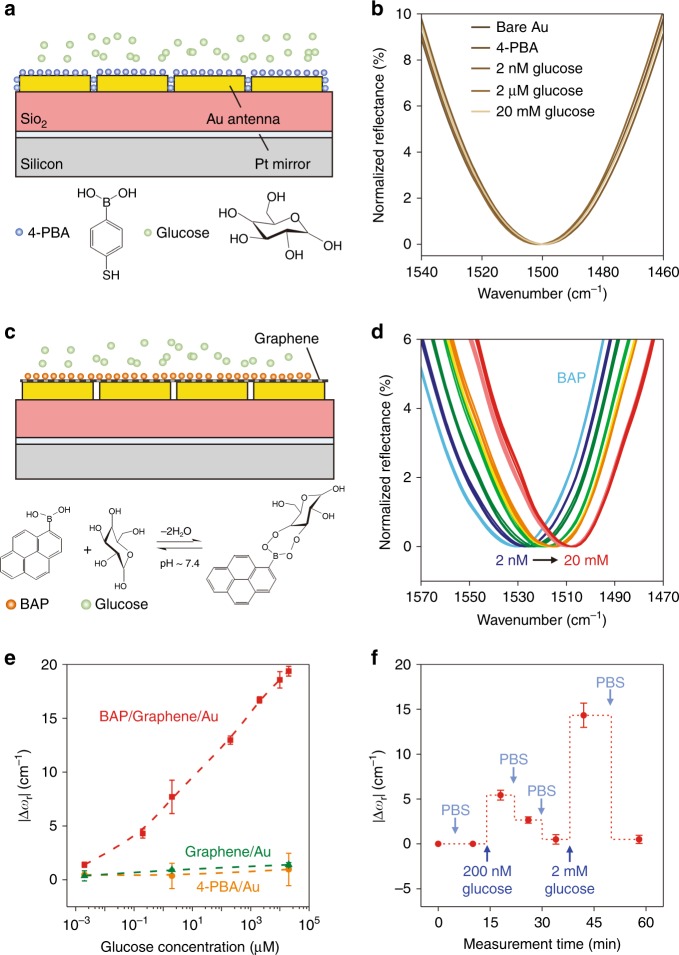
Affinity binding-based measurement of glucose. **a** Schematic of a control device without graphene. **b** Reflectance spectra measured with 4-PBA functionalized control device at different glucose concentrations. **c** Schematic of a hybrid metasurface sensor. **d** Spectral measurement of glucose from 2 to 20 mM on the hybrid metasurface. For each concentration, three measurements were conducted on the same device. **e** |∆*ω*_r_| as a function of glucose concentration for different sensor configurations. **f** Reversible measurement of glucose. The data points were achieved from ex-situ measurements after rinsing the device with each solution. The x-axis reflects the measurement sequence and the time of each solution rinsing. For (**e**)–(**f**), error bars represent standard deviations calculated from nine measurements (three measurements on each of three independent devices)

To first examine the possible responses introduced by refractive index variations due to glucose binding, control experiments were conducted on bare Au metasurfaces treated by 4-mercaptophenylboronic acid (4-PBA) (Fig. 3a). X-ray photoelectron spectroscopy was used to confirm the successful immobilization of 4-PBA on the bare Au metasurface (Figure S7). The shift of *ω*_r_ of the Au metasurface after 4-PBA immobilization followed by exposure to 20 mM glucose solution was merely 1~2 cm^−1^ (Fig. 3b), which corroborated the inability of a monolayer of small molecules to introduce significant variations in local refractive indices.

We then performed measurements on the hybrid metasurface. Without any intended functionalization of graphene, *ω*_r_ did not change after exposure of the device to glucose solutions (Figure S8). In contrast, when the graphene was treated by BAP, *ω*_r_ significantly shifted to lower wavenumbers after exposure to glucose solutions (Figs. 3c, d). This was because the boronic acid, with an empty orbital, initially p-doped graphene by withdrawing electrons from graphene, and this electron-withdrawing capability was weakened upon the binding of glucose (Figure S9). Exposure of the device to 2 nM (0.36 ng/mL) glucose led to a clearly resolvable red-shift of ~1.5 cm^−1^. The detection limit was improved by almost five orders of magnitude compared to the existing affinity binding-based optical glucose sensors that measured the changes in local or bulk refractive indices^46–49^. We note that the high sensitivity does not rely on a specific biochemical process and can be adapted to sense other small molecules, such as gas molecules, since graphene is very sensitive to gas adsorption^34^. Increasing the glucose concentration up to 20 mM monotonically red-shifted the plasmonic resonance. Figure 3e plots the shifts amount of *ω*_r_ for different sensor configurations, which clearly shows that the combination of graphene and the metallic metasurface, with BAP functionalization, led to the most significant sensor responses. The dynamic range of the sensor, extracted from this plot, was 2 to 10 mM, over six orders of magnitude. Fitting of the experimental results to the Hill–Langmuir equation gave an estimated dissociation constant of 99.2 µM (Figure S10). Fig. 3f shows the ex-situ measurements after exposure to high glucose concentrations followed by rinsing with fresh buffer solution. The increase and decrease in |∆*ω*_r_|, observed after rinsing with glucose solutions and fresh buffer solutions, respectively, demonstrated that the sensor was able to detect both increases and decreases in glucose concentrations thanks to the reversibility of the glucose-boronic acid binding.

### Influence of antenna geometry on sensitivity

**Fig. 4:**
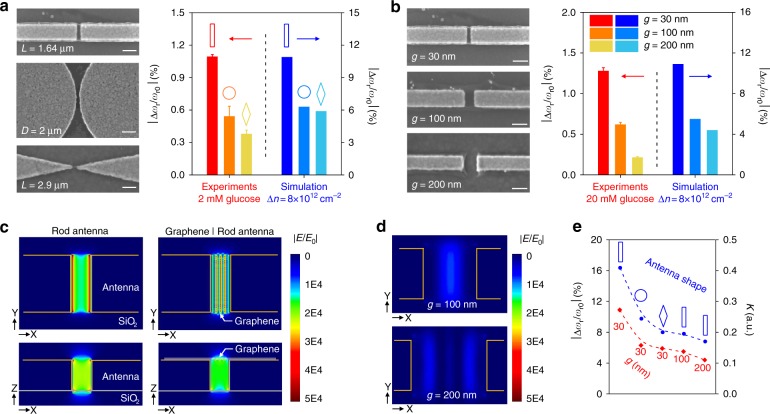
Dependence of metasurface sensitivity on antenna geometry. **a** Left: SEM images of rod, disk, and diamond-shaped antennas. Gap size *g* *=* 30 nm. Scale bars: 100 nm. Right: Relative resonance shifts obtained with different antenna geometries. **b** Left: SEM images of rod antennas with *g* *=* 30, 100, and 200 nm. Scale bars: 200 nm. Right: Relative resonance shifts obtained with different gap sizes. Error bars represent standard deviations from three measurements on three independent devices. **c** Near-field simulation for rod antenna. Left: Bare Au antennas. Right: Graphene-coated Au antennas. The graphene carrier density is 8 × 10^12^ cm^−2^. **d** Simulated near-fields for graphene-coated rod antennas with *g* *=* 100 and 200 nm. **e** Comparison of the trends of |∆*ω*_r_/*ω*_r0_| and *K* for different antenna geometries

We further investigated the influence of the antenna’s geometry on its biosensing sensitivity. First, with a fixed 30- nm gap size between the neighboring antennas, we conducted studies to compare the sensitivities of rod, disk, and diamond-shaped antennas (Fig. 4a). The dimensions of these antennas were chosen to ensure that the initial resonance frequencies, *ω*_r0_, of the metasurfaces (i.e., antenna arrays with bare graphene) were close to each other. The relative shifts of the plasmonic resonance, defined as ∆*ω*_r_ normalized by *ω*_r0_, were used to measure sensitivity. After exposure to 2 mM glucose solution, ∆*ω*_r_/∆*ω*_r0_ were 0.54 and 0.38% for disk and diamond-shaped antennas, respectively (Fig. 4a), which were reduced by 50.4 and 65.1%, compared to that achieved using the rod antennas. FDTD simulation was performed based on a larger modulation of graphene carrier density of 8 × 10^12^ cm^−2^ to achieve more readily recognizable shifts of ∆*ω*_r_. The values of ∆*ω*_r_/∆*ω*_r0_ in our experiments are in good agreement with the simulation results, which predicted that the sensitivities of the disk and diamond-shaped antennas were decreased by 42.2% and 45.1%, respectively, compared to that of the rod antennas. We then fixed the geometry of nanorod antennas and studied the effect of the gap size *g* on the sensitivity. When *g* was increased from 30 to 100 nm and then to 200 nm (Fig. 4b), ∆*ω*_r_/∆*ω*_r0_ introduced by 20 mM glucose decreased by nearly 50% and 80%, respectively, qualitatively agreeing with the simulation results.

To explain the dependence of sensitivity on the antenna geometry, we also calculated the sensitivities for different antenna geometries based on perturbation theory. The equation below relates the sensitivity to the electrical field energy confined by the metasurface (see the Supplementary Information for a detailed derivation of this equation):

1 $$\left| {\frac{{\Delta \omega _r}}{{\omega _{r0}}}} \right| \propto |\Delta \varepsilon |\cdot\left| {\frac{{{\int}{\!\!\int}{\!\!\int} {{\rm{d}}s\left( {\overrightarrow E \cdot \overrightarrow E _0^*} \right)} }}{{{\int}{\!\!\int}{\!\!\int} {{\rm{d}}v} \left( {\varepsilon |\overrightarrow {E_0} |^2} \right)}}} \right| = |\Delta \varepsilon | \cdot K$$

Here, ∆ε represents the change of the local permittivity due to the variation of graphene optical conductivity, *E*_0_ and *E* are, respectively, the electrical fields before and after perturbation (i.e., molecular doping of graphene in this study). *S* in the numerator integration is the area of the graphene suspended over the nanogap, while *V* in the denominator covers all the space where *E*_0_ is non-zero (see Supplementary Information section 5.1 for details). The factor *K* is a variant representing the relative change in electric-field energy distribution, which depends on the antenna geometry and can be calculated from the near-field distributions (Fig. 4c, d and Figure S11). The trends of the theoretically calculated *K* (see the Supplementary Information for details) and the simulated ∆*ω*_r_/*ω*_r0_ agreed nicely with each other for the above five antenna geometries (Fig. 4e). This confirms that the nanorod antennas with 30 nm gaps offer the highest sensitivity. The nanorod antennas show the best performance among the three commonly used antenna geometries because they provide a balance between a strong electric-field enhancement and the volume within which the electric-field can effectively interact with adsorbed molecules on graphene. In contrast, although the diamond antenna shows a higher maximum value of the field enhancement, the ratio of the *E* field confined at the graphene surface relative to the total *E* field confined within antenna system was lower. This can also be found from the cross-section views of the near-field distribution and explains the inferior sensitivity of the diamond antenna. On the other hand, with a given antenna shape, increasing the gap between the adjacent nanorod antennas leads to a linear increase in the perturbation volume but an exponential decrease in the electric-field enhancement and thus will also reduce the sensitivity.

It is noteworthy that the electric-field distribution is changed significantly by adding a monolayer of graphene on the metallic antennas. When graphene is present at the antenna gap, surface plasmon can be excited on graphene surface; thus, standing waves can be formed with multiple maxima located on the suspended graphene, subject to the boundary conditions defined by the metal antenna where the electrical field is zero. In contrast, metallic metasurfaces only support hotspots at the edges of antennas (Fig. 4c). The hotspots distributed along the suspended graphene, despite the slightly reduced maximum enhancement value, create more space for effective light-matter interaction and may particularly benefit measurement at very low concentrations.

**Fig. 5:**
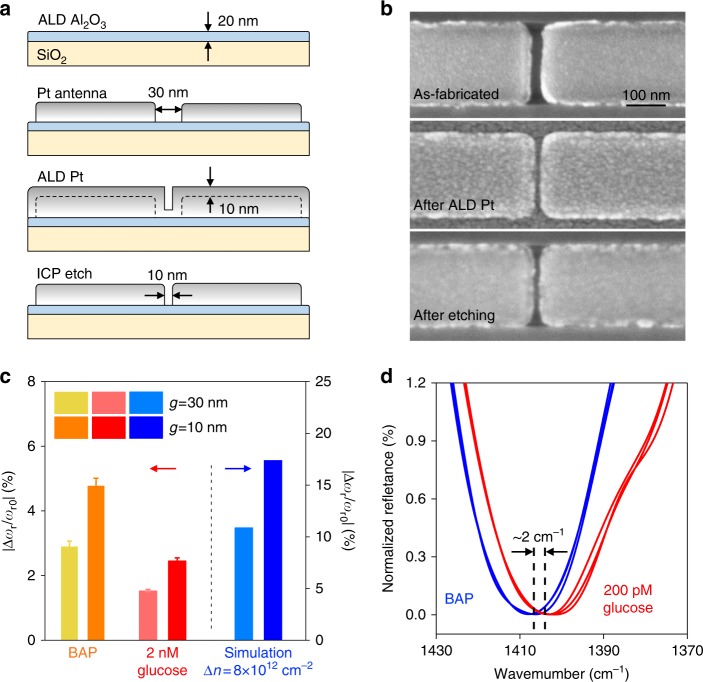
Sensitivity improvement in metasurfaces with 10-nm antenna gaps. **a** Fabrication process to create 10-nm antenna gaps. **b** SEM images of one antenna gap at each step of fabrication. **c** Comparison of sensitivities between the cases of *g* *=* 30 and 10 nm. (**d**) Detection of 200 pM glucose on a metasurface with *g* *=* 10 nm

The monotonic increase of the sensitivity with decreasing antenna gap size predicts that the sensitivity would continue to improve by bringing the neighboring antennas closer to each other. However, reducing the gap size to below 30 nm would be very challenging for standard lithography and lift-off processes considering the high aspect ratio of the nanostructures (antenna width of ~200 nm and thickness of ~50 nm). Therefore, a new method was developed to fabricate antenna arrays with a *g* value of 10 nm (Fig. 5a). First, a layer of Al_2_O_3_ was deposited on the SiO_2_ as a protection layer using atomic layer deposition (ALD). Then, Pt antennas with initial gaps of 30 nm were fabricated and an additional 10-nm Pt layer was homogeneously grown using ALD. Finally, a 10-nm Pt layer was anisotopically etched away using inductively coupled plasma (ICP) to create 10- nm gaps between adjacent antennas (Fig. 5b). BAP functionalization of such antenna arrays with 10-nm gaps generated a blue-shift of *ω*_r_ of 4.77%, which was 64% larger than that achieved by nanorods with 30- nm gaps (Fig. 5c). Exposure of the BAP-activated metasurface to a 2 mM glucose solution introduced a resonance shift of 1.76%, which was also improved by 60% compared to the case of *g* = 30 nm. A low concentration of 200 pM glucose (36 pg/mL) was detectable on our 10- nm gap metasurface (Fig. 5d), which indicates that the detection limit of the sensor was further improved by approximately one order of magnitude compared to previously demonstrated 30-nm gap metasurfaces. To compare with typical surface plasmon resonance biosensors, we also estimated the detection limit in weight per unit area (pg/mm^2^), which is an important figure of merit for surface-based sensors. In the specific case of glucose sensing, the detection limit was approximately 1.3 pg/mm^2^ when the gap was 30 nm, and further lowered to 0.043 pg/mm^2^ when the gap was 10 nm, which was three times better than that for the state-of-the-art Vernier effect plasmonic resonance biosensors (see Supplementary Information section 5.3 for details on the calculation of the detection limit)^50^. However, these values should not represent the theoretical limit of our method, considering that the optical conductivity change is mainly dependent on the number of charges transferred to graphene rather than the molecular weights of the adsorbed molecules. Therefore, this limit value can be even lower for the detection of stronger dopants with lower molecular weights. It can also be improved by engineering the antenna geometry. Figure 4e implies that the ultimate limit of detection for our approach is defined by quantum effects. Briefly, when the gap size is aggressively reduced to ~0.3 nm, direct electron tunneling between the neighboring antenna will cause a sharp decrease in the field intensity within the gap; thus, the sensitivity will no longer increase^51,52^.

### Enhancement of molecular vibrational fingerprints

**Fig. 6:**
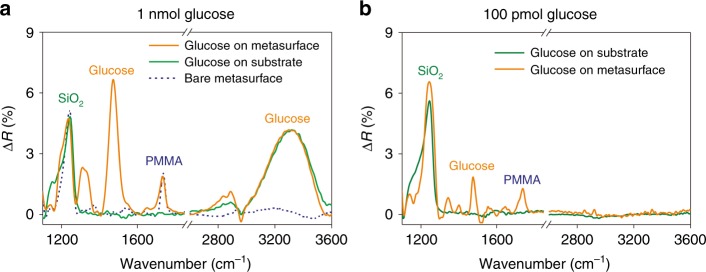
Enhancement of glucose fingerprints. Optical absorption (∆R) measured on metallic metasurface without graphene before and after drop-coating. **a** 1 nmole and **b** 100 pmole of glucose on a gold metasurface device with 30-nm antenna gaps

The metasurface can also act as a substrate for surface-enhanced IR absorption (SEIRA) spectroscopy. Here, we qualitatively examined the enhancement effect on the glucose fingerprints located within 1300–1500 cm^−1^, which typically show weaker absorption than glucose OH groups do near 3000 cm^−1^. We first drop-coated, in total, 1 nmol (2 mM, 0.5 µL) of glucose on a metallic metasurface device. In measurements performed on the bare substrate (SiO_2_-Pt cavity on Si) out of the metasurface region, we found that when the absorption of the OH band was 4.2% (see the Supplementary Information for details), other bands of glucose were hardly recognizable (Fig. 6a). In contrast, for measurements performed within the metasurface (*g* = 30 nm), when the intensity of the OH band was close to that measured on bare substrate, the spectrum revealed prominent peaks at 1470 and 1320 cm^−1^, which were attributed to glucose OCH deformation. The enhancement effect was also observed at 1740 cm^−1^, which corresponded to the C=O bond of the polymethyl methacrylate (PMMA) residue from the fabrication. Further, when the glucose quantity was reduced to 100 pmol (200 µM, 0.5 µL), the OCH band at 1470 cm^−1^ was still recognizable (Fig. 6b) with a signal-to-noise ratio of 39, even if there was essentially no signal from the OH band as it fell out of the metasurface resonance band. These results demonstrated that the metasurface provided remarkable enhancement of the vibrational fingerprints, which originated from the enhanced light-matter interaction by the strongly confined electrical field at the nanogap and the edges of the metallic antenna. Such fingerprinting enhancement can help identify analytes in biochemical sensing, especially when the analyte quantity is very limited.

## Discussion

In summary, the hybrid graphene-metallic metasurface enables ultrasensitive optical measurements of small molecules as well as an enhancement of their fingerprints. The optical conductivity-based sensing shows detection limits orders of magnitude lower than conventional optical sensing techniques based on local refractive index changes. It also allows for differentiation between low-molecular-weight analytes based on different types and/or concentrations of dopants introduced to graphene.

While this paper is focused on demonstrating the principle of this new sensing approach by performing measurements in air, the use of such a sensor is not limited to dry environments and can be further complemented. For example, it would be possible to make the device mid-IR transparent by replacing SiO_2_ with CaF_2_ and removing the Pt layer, which may allow for integration of the sensor with microfluidic channels for on-chip real-time sensing or monitoring of the binding process^33^. To enable the application of the sensor in complex physiological fluids, it is important to develop a functionalization process of graphene to reject non-specific absorption, which is a challenge in general for biosensors employing nanomaterials. In addition, the long-term stability and consistency of sensor output needs to be further studied and improved, possibly by protection of the graphene surface and enhancement of graphene-substrate adhesion^53,54^.

## Materials and methods

### *Device fabrication*

(a) Fabrication of the hybrid graphene-metallic metasurfaces. A 50-nm Pt layer was deposited on a heavily doped silicon substrate, followed by deposition of a 285-nm SiO_2_ layer using plasma-enhanced chemical vapor deposition (PECVD). Gold nanorods 50 nm in thickness were then fabricated over a 400 µm by 400 µm area on the SiO_2_ layer using electron beam lithography, electron beam evaporation and lift-off processes. A monolayer graphene film grown on a copper foil via chemical vapor deposition (CVD) was then transferred onto the gold nanorod array using a PMMA-assisted wet transfer method. The PMMA layer was lastly removed by rinsing the device in acetone for over 3 h at room temperature. (b) Fabrication of graphene FETs. A CVD graphene film was transferred onto a thermally oxidized (285-nm SiO_2_) silicon substrate, and metallic contacts (2-nm Ti and 45-nm Au) were patterned using electron beam lithography, A monolayer graphene film grown on a copper foil via chemical vapor deposition (CVD) was then transferred onto the gold nanorod array using a PMMA-assisted wet transfer method. The PMMA layer was lastly removed by rinsing the device in acetone for over 3h at room temperature. (b) Fabrication of graphene FETs. A CVD graphene film was transferred onto a thermally oxidized (285-nm SiO) silicon substrate, and metallic contacts (2-nm Ti and 45-nm Au) were patterned using electron beam lithography, electron beam evaporation and lift-off processes.electron beam evaporation and lift-off processes.

### *Biochemical functionalization*

(a) Immobilization of pyrene derivatives on graphene. 0.5 mM amino-pyrene (AP) or boronic acid-pyrene (BAP) solutions were prepared by dissolving the powders in anhydrous acetonitrile (Sigma-Aldrich). Metasurface devices with as-transferred graphene were immersed in a 2 mL AP or BAP solution for 12 h, and then rinsed with fresh acetonitrile, isopropanol and deionized water baths sequentially to remove molecules that were not immobilized on the graphene. The devices were then gently blow-dried using compressed nitrogen and left in vacuum desiccator for over 30 min at room temperature to completely remove solvents without impacting the properties of graphene. To immobilize boronic acid on gold antennas, devices with bare gold nanorods were immersed in 4-mercaptophenylboronic acid (Fisher Scientific) solution (1 mM in ethanol) for 12 h at room temperature, and then rinsed with fresh ethanol and deionized water baths sequentially. (b) Aluminum nanoparticles were deposited using thermal evaporation. The devices with graphene and the control devices without graphene were secured on a same glass slide for deposition under the same conditions (0.1 A/s). (c) Glucose solutions were prepared by dissolving D-glucose (Sigma-Aldrich) in 1 × phosphate buffered solutions (ThermoFisher Scientific) and diluted to desired concentrations. At each concentration, the device was exposed to glucose solution for 8 min to ensure that the glucose-boronic acid binding reached equilibrium. The devices were then dipped in fresh buffer and ultrapure water (ThermoFisher) and gently blow-dried using purified nitrogen.

### *Material characterization*

The hybrid graphene-metallic metasurfaces and deposited Al nanoparticles were characterized using a scanning electron microscope (SEM, Zeiss Sigma VP). Characterization of the functionalized graphene was conducted using Raman spectroscopy (Renishaw) with an exciting laser at λ = 532 nm. Functionalization of gold nanorods with 4-PBA was characterized using X-ray photoelectron spectroscopy (XPS, Phi 5500). The thicknesses of the graphene before and after chemical treatment were measured using an atomic force microscope (AFM, Bruker Dimension FastScan).

### *Measurements setup*

(a) Electrical measurements: Transport characteristics of graphene were measured using Keithley 2400 source-meters. (b) Optical measurements: Infrared reflectance spectra of metasurfaces were measured using a Fourier transform infrared (FTIR) microscope (Bruker Vertex 70 v) with light polarized along the antenna rods. A spectrum measured on a gold mirror was used as the reference for reflectance spectral measurements. Measurements at different glucose concentrations were carried out on the same metasurface from the same location with a window size as large as the metasurface area (400 µm by 400 µm). All spectral measurements were carried out in ambient atmosphere at room temperature.

## Electronic supplementary material

Supplementary information for Optical Conductivity-Based Ultrasensitive Mid-Infrared Biosensing on a Hybrid Metasurface
