# Supplementary material for: Optical conductivity-based ultrasensitive mid-infrared biosensing on a hybrid metasurface
Source: Light Sci Appl. 2018 Sep 26;7:67. doi: 10.1038/s41377-018-0066-1 (PMC6156330; doi:10.1038/s41377-018-0066-1)
Supplement: Supplementary file 1 — Supplementary information for Optical Conductivity-Based Ultrasensitive Mid-Infrared Biosensing on a Hybrid Metasurface [file 41377_2018_66_MOESM1_ESM.docx]

Supplementary information for

**Optical Conductivity-Based Ultrasensitive Mid-Infrared Biosensing**

**on a Hybrid Metasurface**

Yibo Zhu^†^, Zhaoyi Li^†^, Zhuang Hao, Christopher DiMarco, Panita Maturavongsadit, Yufeng Hao, Ming Lu, Aaron Stein, Qian Wang, James Hone, Nanfang Yu^*^, and Qiao Lin^*^

^1^Department of Mechanical Engineering, Columbia University, New York, NY, 10027, United States.

^2^Department of Applied Physics and Applied Math, Columbia University, New York, NY, 10027, United States.

^3^Department of Chemistry and Biochemistry, University of South Carolina, Columbia, SC, 29208, United States.

^4^National Laboratory of Solid State Microstructures, College of Engineering and Applied Sciences, and Collaborative Innovation Center of Advanced Microstructures, Nanjing University, Nanjing 210093, China.

^5^Center for Functional Nanomaterials, Brookhaven National Laboratory, Upton, NY 11973, USA

^†^These authors contributed equally to this work.

^*^Emails: ny2214@columbia.edu (N.Y.), qlin@columbia.edu (Q.L.).

**1. Characterization of graphene**

1.1 Microscopy and spectroscopy characterization of graphene

The graphene used in this work was grown on Cu substrate using chemical vapor deposition (CVD) and transferred using wet-transfer method. Figure S1a shows a optical microscope image of the graphene transferred on Si substrate with 285 nm thermal oxide layer. Based on the color contrast given by different layers of graphene, most of the graphene was monolayer while there were add-on layers at sparse local spots which would not significantly impact our device performance.


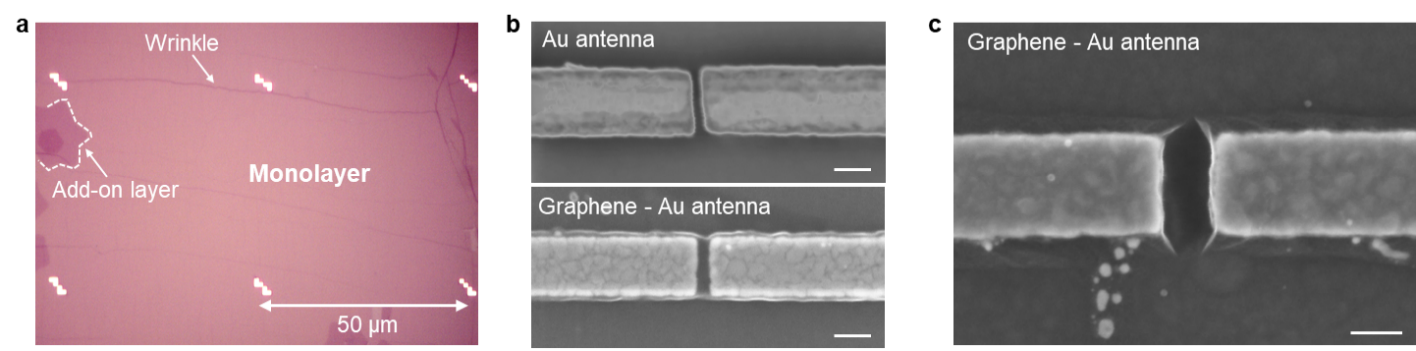


**Figure S1.** Microscopic characterization of graphene. (a) Optical microscope photo of CVD graphene transferred on 285 nm SiO_2_/Si substrate with prepatterned Au alignment marks. (b) Scanning electron microscope (SEM) images of Au antennas with 30 nm gap before (upper) and after (lower) graphene transfer. (c) SEM image of graphene-covered Au antennas with 100 nm gap. Scale bars in (b) and (c): 100 nm. Both (b) and (c) clearly show the presence of graphene and indicate that graphene conformed to the antennas.

1.2 Characterization of chemical treatment with graphene

Raman spectroscopy of graphene treated by AP and BAP solutions showed peaks corresponding to NH_2_ (1190 cm^-1^) and BOH (1286 cm^-1^), respectively; the peaks corresponding to pyrene groups (1407, 1620 cm^-1^) were also observed on both samples, indicating that the pyrene derivatives had been successfully immobilized on graphene. There were no additional peaks observed on the ACN-rinsed graphene other than G and 2D bands, indicating that the chemical treatment did not have detrimental effects on the crystal structure of graphene. However, the broadening of the 2D band suggested that the carrier mobility of graphene had decreased. Atomic force microscopy (AFM) revealed that the thickness of graphene increased by approximately 0.5 nm after functionalization of BAP molecules, which was in agreement with reported values^1^.


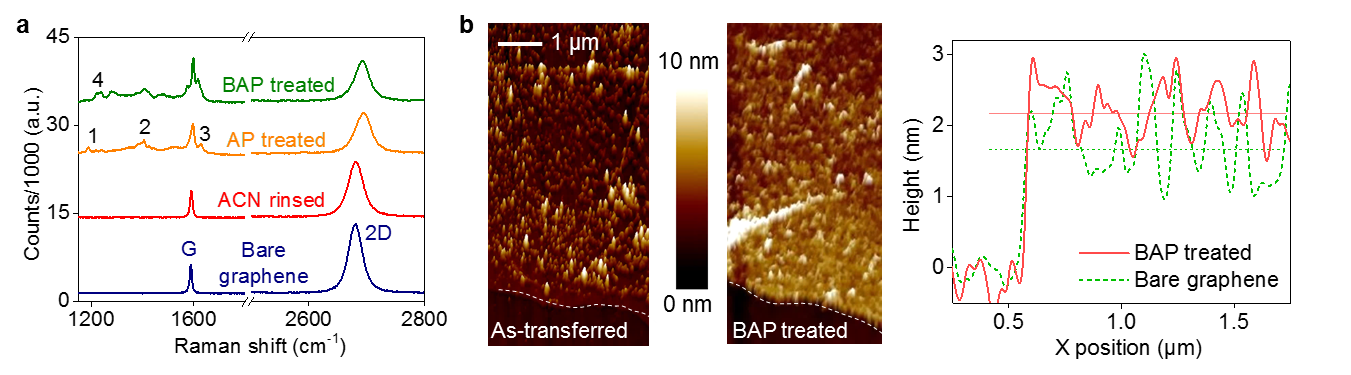


**Figure S2.** Characterization of graphene functionalized by biochemicals. (a) Raman spectroscopy of graphene treated by different chemicals. Position of peaks: #1. 1190 cm^-1^, #2. 1407 cm^-1^, #3. 1620 cm^-1^ and #4. 1286 cm^-1^. (b) Atomic force microscopy (AFM) studies. Left: morphology of graphene before and after BAP immobilization. Right: Thickness change before and after BAP immobilization, showing an increase of 0.5 nm in height, which can be considered as the thickness of the BAP molecules.

1.2. Electrical characterization

Graphene carrier density was calculated by multiplying the gate capacitance *C_G_* (11.8 nF∙cm^2^) with the Dirac point voltage (V_NP_), at which the resistance of graphene reached its maximum. The graphene carrier mobility *µ* was calculated from the transconductance of the transport characteristics.


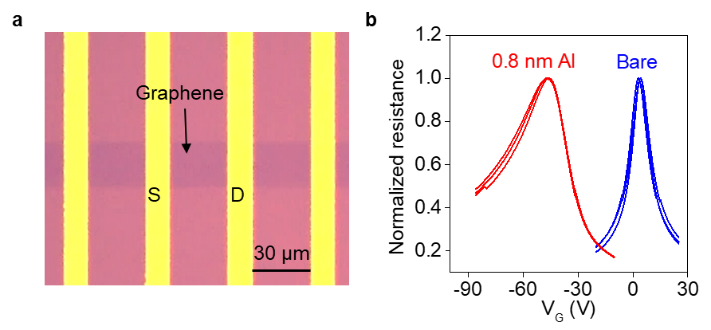


**Figure S3.** Measurement of graphene FET transport characteristics. (a) Optical microscope of graphene FET. (b) Transport characteristics measured before and after deposition of 0.8 nm Al particles. The capacitor consisted of 285 nm thick SiO_2_.

**2. FDTD simulation**

2.1 Simulation setup

The simulation was performed using the Lumerical FDTD (Finite-Difference Time-Domain) software. In the simulation, the complex optical refractive indices of gold and silicon dioxide were obtained from Palik database^2^. The incident light was a linearly polarized plane wave with its polarization direction along the antenna rods.

2.2 Graphene optical conductivity model

In the simulation, the graphene was modeled as an anisotropic material and its thickness was set to be 0.33nm. Its out-of-plane optical refractive indices were set to be n=2.5 and k=0. Its in-plane refractive indices were calculated based on its in-plane optical conductivity that was derived using random phase approximation^3^:

where *e* is the elementary charge, *k_B_* is the Boltzmann constant, *T* is temperature, is the reduced Planck constant, and *τ* is carrier relaxation lifetime that characterizes electron-disorder scattering processes; is the Fermi energy, where *n* is the carrier doping level and *V_f_* =10^6^ m/s is the Fermi velocity. The carrier mobility is$\mu=\frac{\tau e}{m_{eff}}$, where the effective mass is $m_{eff}=\frac{E_{f}}{V_{f}^{2}}$. The first term in the equation above is contributed by intraband transition and the second term corresponds to interband transition.

2.3 Metasurface spectral property at different graphene carrier mobilities

In agreement with experimental results and the results of the equivalent circuit model, the FDTD simulations showed that changes in graphene carrier mobility did not significantly vary the Q factor of the metasurface resonance. This indicated that the sensor is insensitive to the degradation of carrier mobility and therefore is robust for biochemical applications.


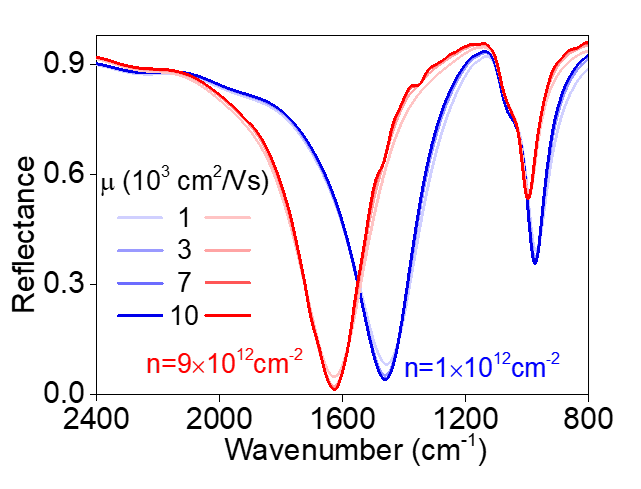


**Figure S4.** FDTD simulations of the metasurface resonance at different values of graphene carrier density and mobility.

**3. Equivalent circuit model of the hybrid metasurface**

3.1 Parameter setup

In the circuit model, the initial values of R_A_, C_A_, R_M_, L_M_, and C_M_ were adapted from ref. 4 and the graphene inductance L_G_ and resistance R_G_ were determined by theoretical calculations (Figure S5), which are described in section 3.2. To study the effects of graphene carrier mobility, L_G_ and R_G_ were calculated at λ=7 µm (i.e., 1430 cm^-1^), which was close to the plasmon resonance frequency. The parameter values used in the circuit model at different graphene carrier mobilities are listed in table S1.


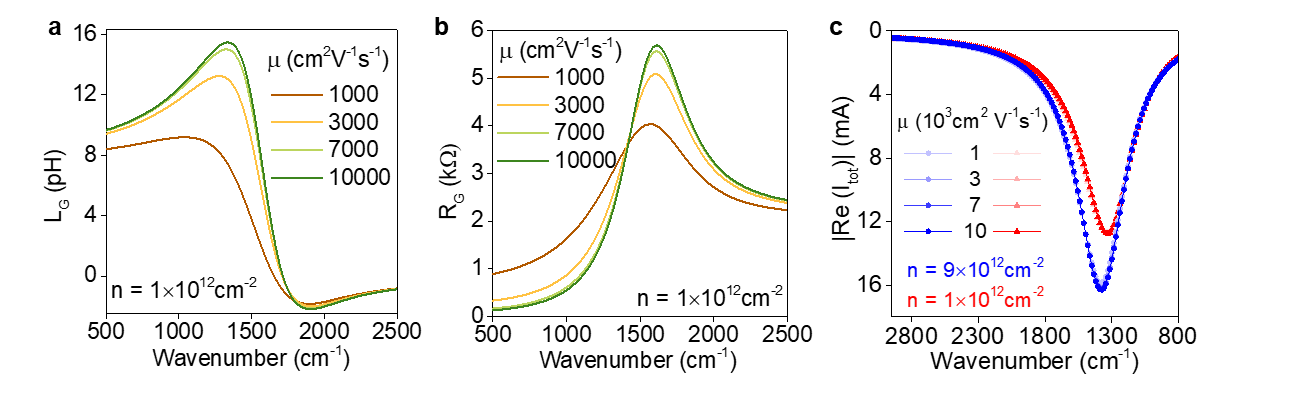


**Figure S5.** Calculations of the effective graphene inductance and resistance in the circuit model. Frequency dependent graphene load (a) inductance L_G_ and (b) resistance R_G_ at n=1×10^12^ cm^-2^ and different mobilities. (c) Frequency response of the current I_tot_ in the equivalent circuit model at different graphene carrier density and mobility levels.

**Table S1.** Parameter values used in the equivalent circuit model

| V_in_ (V) | R_A0_ (Ω) | C_A0_ (F) | R_M0_ (Ω) | L_M0_ (pH) | C_M0_ (F) |
| --- | --- | --- | --- | --- | --- |
| 100 | 1k | 0.0321f | 63 | 0.92 | 0.0384f |

| Graphene carrier density n=1×10^12^cm^-2^ | | |  | Graphene carrier density n=9×10^12^cm^-2^ | | |
| --- | --- | --- | --- | --- | --- | --- |
| µ (cm^2^V^-1^s^-1^) | R_G_ (Ω) | L_G_ (pH) |  | µ (cm^2^V^-1^s^-1^) | R_G_ (Ω) | L_G_ (pH) |
| 1000 | 3900 | 3.7 |  | 1000 | 117 | 3.3 |
| 3000 | 4500 | 8.9 |  | 3000 | 48 | 3.3 |
| 7000 | 4800 | 11.2 |  | 7000 | 28 | 3.3 |
| 10000 | 4900 | 11.7 |  | 10000 | 24 | 3.3 |

3.2 Calculations of L_G_ and R_G_

The in-plane electrical permittivity of graphene can be derived based on its in-plane optical conductivity using the following equation:

$$\varepsilon(\omega)=2.5+i\frac{\sigma(\omega)}{\varepsilon_{0}t\omega}$$

where 2.5 is permittivity at the infinite frequency, $\varepsilon_{0}$ is the vacuum permittivity, and *t* is the graphene thickness (0.33 nm). Then, the impedance of graphene can be obtained as:

$$Z_{G}=\frac{l}{-i\omega\varepsilon\varepsilon_{0}wt}$$

where *l* is the antenna gap size, and *w* is the width of rod antenna. The real part of it is graphene resistance R_G_, and the imaginary part of it is graphene reactance X_G_. The inductance of graphene L_G_ is related with X_G_ via $L_{G}=\frac{X_{G}}{\omega}$ .

3.3 Influence of antenna impedance and capacitance

The antenna impedance Z_M_ = R_M_ + jωL_M_ used in the circuit model should be a function of the properties of the metallic antennas such as their geometry and the conductivity of the metal. To study how Z_M_ can influence ω_r_ and the Q factor, we varied Z_M_ by increasing or decreasing R_M_ and L_M_ at the same rate with respect to the initial values R_M0_ and L_M0_, to maintain the phase of Z_M_. Compared to varying graphene carrier mobility (Figure 2), variations of Z_M_ lead to more significant changes of the metasurface Q factor (Figure S6). This suggests that the Q factor is mainly determined by the metallic antennas.

On the other hand, the antenna capacitance (C_M_) is a function of the permittivity of the medium in the gap between a pair of neighboring antennas. For typical plasmonic sensors based on changes of local refractive indices, adsorption of molecules on the surface of the metal antennas changes the permittivity of the gap medium. In our circuit model, 12% increase in C_M_ caused a red-shift of the resonance frequency of 33 cm^-1^. This value was comparable or even less than the shifts caused by monolayer small molecules on the graphene-metallic hybrid metasurface. This suggests that the modulation of graphene conductivity using monolayer small molecules is equivalent to a change of the gap permittivity by more than 12%. Therefore, our optical conductivity-based sensor offers a more sensitive solution for plasmonic biosensing.

**Figure S6.** Influence of the antenna impedance on the metasurface Q factor. (a) Spectral response calculated using the circuit model at different values of antenna impedance (Z_M_ = R_M_ + jωL_M_ without changing capacitance). (b) Variations of the Q factor as a function of the antenna impedance. (c) Red-shifts of the metasurface resonance caused by the increase of the antenna capacitance C_M_.

3.4 Influence of graphene carrier density and mobility

For graphene load, the impedance is a function of graphene resistance and inductance. As shown in table S1 in SI, given a same carrier density, when the mobility is varied by 10 times from 1000 cm^2^V^-1^s^-1^ to 10000 cm^2^V^-1^s^-1^, the inductance or resistance of graphene only shows 3 to 5 folds change. In contrast, when the carrier density is varied by 9 times from 1×10^13^ cm^-2^ to 9×10^13^ cm^-2^, the resistance can be changed by at least 33 times, up to 204 times (at mobility of 10000 cm^2^V^-1^s^-1^). This indicates that the impedance of the graphene load is significantly (more than one order of magnitudes) more sensitive to the carrier density than to the carrier mobility.

**4. Glucose measurements**

4.1 Control experiments on a bare Au metasurface

**Figure S7.** X-ray photoelectron spectroscopy characterization of Au antennas functionalized with 4-PBA. The 4-PBA treated Au surface showed peaks attributable to sulfur and boron.

4.2 Control experiments on a bare graphene sheet


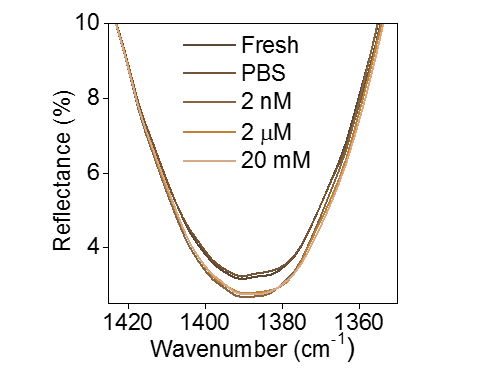


**Figure S8.** Control experiments on a unfunctionalized hybrid metasurface (with bare graphene). There was no shift of the resonance frequency by exposing a hybrid metasurface with bare graphene to glucose solutions of different concentrations.

4.2 Change of graphene carrier densities by boronic acid and glucose binding

The increase or decrease in hole density is due to a molecular doping effect when boronic acid is immobilized on graphene. Most boronic acid motifs on the BAP molecules (pKa 8.8) were neutral with an sp^2^ hybridized electron configuration at pH=7.4. Upon anchoring on the surface of graphene, the empty p-orbital of boron generates a strong electron withdrawing effect by depriving electrons from graphene, resulting in a p-doping effect. When exposed to glucose solutions, boronate esters will be formed, which favor the equilibrium towards an electron-rich sp^3^ hybridization, thereby generating n-type doping, in agreement with the FET measurement results (Figure S9).

**Figure S9.** Transport characteristics of the graphene FET after BAP functionalization and exposure to 20 mM glucose solutions.

4.3 Fitting of measured |∆ω_r_|

The measured |∆ω_r_| as a function of the glucose concentration was fitted to the Hill-Langmuir equation:

$$\text{|∆}\text{ω}_{r}\text{| }\text{= }\text{A}\frac{\left( \frac{\text{c}}{\text{K}_{\text{d}}} \right)^{\text{n}}}{\text{1+}\left( \frac{\text{c}}{\text{K}_{\text{d}}} \right)^{\text{n}}}$$

where *A* is the sensor saturation response when all boronic acid sites are occupied, *c* is the glucose concentration, *K*_d_ is the dissociation constant for the binding between glucose and boronic acid, and *n* is the Hill coefficient describing the binding cooperativity.

**Figure S10.** Langmuir fitting of |∆ω_r_| in glucose measurement on a hybrid metasurface.

A best fitting (Figure S10) yields *K*_D_ of 99.15 µM, *A* of 24.78, and *n* of 0.238. The Hill coefficient, less than 1, indicates a negative cooperativity of the BAP and glucose binding on the graphene surface^5^.

**5. Dependence of sensitivity on antenna geometry**

5.1 Mathematic expression of ∆ω_r_/ω_r0_

The relative resonance frequency shift of metasurface, ∆ω_r_/ω_r0_, can be calculated by the perturbation theory^6^. It is equal to the ratio of the variation of the electromagnetic energy in the perturbed area and the total electromagnetic energy of the system:

$$\frac{\Delta\omega_{r}}{\omega_{r0}}=\frac{-\iiint dV\left[\left(\Delta\overleftrightarrow{\mu}\cdot\overrightarrow{H}\right)\cdot\overrightarrow{H_{0}}^{*}+\left(\Delta\overleftrightarrow{\varepsilon}\cdot\overrightarrow{E}\right)\cdot\overrightarrow{E_{0}}^{*}\right]}{\iiint dV(\mu\left|\overrightarrow{H_{0}}\right|^{2}+\varepsilon\left|\overrightarrow{E_{0}}\right|^{2})}$$

where $\overrightarrow{E_{0}}$ and $\overrightarrow{H_{0}}$ are the electric and magnetic fields before perturbation, respectively, $\overrightarrow{E}$and$\overrightarrow{H}$ are the electric and magnetic fields after perturbation, respectively. $\Delta\overleftrightarrow{\varepsilon}$ and$\Delta\overleftrightarrow{\mu}$ are the perturbation tensor of permittivity and permeability, respectively. Since graphene only introduces an electrical perturbation (i.e.,$\Delta\overleftrightarrow{\varepsilon}\neq0$ and$\Delta\overleftrightarrow{\mu}=0$), the above expression can be simplified as:

$\frac{\Delta\omega_{r}}{\omega_{r0}}=\frac{-\iiint dV\left[\left(\Delta\overleftrightarrow{\varepsilon}\cdot\overrightarrow{E}\right)\cdot\overrightarrow{E_{0}}^{*}\right]}{\iiint dV(\mu\left|\overrightarrow{H_{0}}\right|^{2}+\varepsilon\left|\overrightarrow{E_{0}}\right|^{2})}$.

Graphene is very thin, so we assume the permittivity perturbation tensor is uniform along its thickness direction. Then the above expression can be rewritten as:

$\frac{\Delta\omega_{r}}{\omega_{r0}}=\frac{-\iint dS\left[\left(\Delta\overleftrightarrow{\varepsilon}\cdot\overrightarrow{E}\right)\cdot\overrightarrow{E_{0}}^{*}\right]\cdot t_{g}}{\iiint dV(\mu\left|\overrightarrow{H_{0}}\right|^{2}+\varepsilon\left|\overrightarrow{E_{0}}\right|^{2})}$.

Furthermore, assuming that the in-plane permittivity tensor of graphene is isotropic, the above expression can be further rewritten as:

$\left|\frac{\Delta\omega_{r}}{\omega_{r0}}\right|\approx\left|\frac{\iint dS\left[\left(\Delta\overleftrightarrow{\varepsilon}\cdot\overrightarrow{E}\right)\cdot\overrightarrow{E_{0}}^{*}\right]\cdot t_{g}}{\iiint dV\left(\mu\left|\overrightarrow{H_{0}}\right|^{2}+\varepsilon\left|\overrightarrow{E_{0}}\right|^{2}\right)}\right|\propto\left|\Delta\varepsilon\right|\cdot\left|\frac{\iint dS(\overrightarrow{E}\cdot\overrightarrow{E_{0}}^{*})}{\iiint dV\left(\mu\left|\overrightarrow{H_{0}}\right|^{2}+\varepsilon\left|\overrightarrow{E_{0}}\right|^{2}\right)}\right|$.

The first term, ∆ε, represents the change of the local permittivity due to the variation of graphene optical conductivity, which is a function of carrier doping to graphene. The second term is the relative change of electric energy distribution, which is determined by the antenna geometry and can be calculated from simulated electric near-field distributions. We define the second term in the above expression as a K factor and obtained its value from full-wave simulations.

$$K=\left|\frac{\iint dS(\overrightarrow{E}\cdot\overrightarrow{E_{0}}^{*})}{\iiint dV\left(\mu\left|\overrightarrow{H_{0}}\right|^{2}+\varepsilon\left|\overrightarrow{E_{0}}\right|^{2}\right)}\right|$$

We note that *S* in the numerator is the area of the graphene suspended over the nanogap, while *V* in the denominator covers all the space where E_0_ is non-zero. To justify this, ∆ε is non-zero only over the 2D graphene monolayer due to the change of graphene optical conductivity, and E is non-zero only in the nanogap as indicated by the near field simulation. Therefore, the product of ∆ε and E is non-zero only at the monolayer graphene surface suspended over the nanogap. However, there is strong electrical field concentrated at the bottom corners of the antennas, which does not contribute to the numerator integration. From the near field simulation, it can be found (both visually and quantitatively) that for diamond antennas the percentage of the field energy confined at the bottom corner is higher than that in rod and disk antennas, thus the sensitivity of the diamond antenna was lower.

5.2 Calculations of variant *K* from simulated near-fields

For each antenna geometry, we conducted a pair of simulations: one with graphene covering the metasurface and the other without graphene. Other than graphene, the simulation setup is kept the same. We monitored the in-plane electric field component at the interface between graphene and metasurface in two simulations at their resonance frequencies, and integraated the product of $\overrightarrow{E}$ and $\overrightarrow{E_{0}}^{*}$ over the simulation area, which gives the value of $\left|\iint dS(\overrightarrow{E}\cdot\overrightarrow{E_{0}}^{*})\right|$. Because the metasurface is nearly a perfect absorber at the resonance frequency, we assume that the incident power is totally confined by the metasurface. Thus, we use the incident source power as $\left|\iiint dV\left(\mu\left|\overrightarrow{H_{0}}\right|^{2}+\varepsilon\left|\overrightarrow{E_{0}}\right|^{2}\right)\right|$, and K was obtained by taking the ratio of these two values.


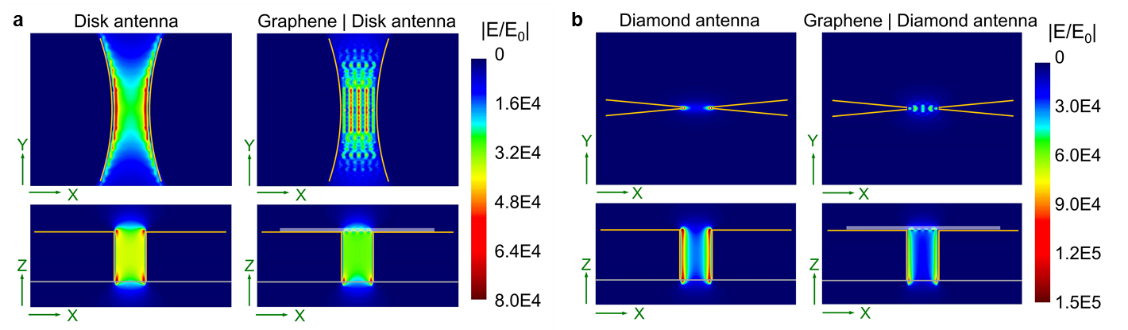


**Figure S11.** Simulated near-fields around antenna gaps for disk and diamond antennas.

5.3 Detection limit estimation

To calculate the detection limit in pg/mm^2^ which is a general figure of merit for surface-based biosensor, it is noteworthy that our device is different from typical surface sensors such as surface plasmon resonance sensor, optical fiber sensor or field effect sensor. In those sensors, the effective sensing area is equivalent to the device area functionalized with receptor molecules. In contrast, the output of our sensor only comes from the binding events at the local, nanometers gap between two antennas, despite that the entire device surface is coated with graphene. Therefore, in calculation of detection limit, it is supposed to only consider the molecules binding to the graphene suspended over the gap. Specifically, in glucose sensing, the density of the pyrene-boronic acid packed on the graphene is around 6000/μm^1^. Thus, for the sensor with 30 nm gap, there would be in total 2.16×10^5^ (3.59×10^-19^ mol) pyrene-boronic acid molecules adsorbed on the graphene over the gap.

In our control experiments we found the readout of the resonance frequency at 2 nM glucose slightly varied from device to device by 0.36 cm^-1^ (Figure 3e). Therefore, the smallest resonance shift we could confidently resolve would be approximately 1 cm^-1^ (three times the 0.36 cm^-1^); and 2 nM glucose (introducing a resonance shift of 1.4 cm^-1^) was close to the lowest concentration we were able to measure using the 30 nm gap device. Given that the glucose binding follows Langmuir isotherm, the coverage of glucose at 2 nM can be calculated by comparing the resonance shift at 2 nM glucose (1.4 cm^-1^) and at 20 mM glucose (19.4 cm^-1^, binding saturated), which is 7%, i.e. 2.5×10^-20^ mol or 4.5×10^-18^ g. The total area taken by each antenna unit (including one gold nanorod plus the space between neighboring nanorods in both x and y directions) was 3.4 μm^2^. Therefore, for the 30 nm gap device, the detection limit for glucose sensing is 1.3 pg/mm^2^. For the 10 nm gap device which can resolve 200 pM glucose, the detection limit is lowered to 0.043 pg/mm^2^, three time better than the state-of-the-art Vernier effect biosensors^7^. This, however, is not the theoretical detection limit of our method. While we selected glucose as a representative analyte for proof of principle, our sensing mechanism indicates that the detection limit is not dependent on molecular mass, but mainly affected by the antenna geometry and charge transfer efficiency to graphene.

**6. Enhancement of fingerprints**

6.1 Extraction of spectral fingerprints

The spectral fingerprints of glucose and PMMA, ∆R, were obtained by subtracting the background reflection spectrum, R_0_, from the measured reflectance spectrum, R. Here, R_0_ is a fit to the metasurface resonance. To obtain R_0_, a few spectral points were selected from a measured spectrum outside the regions where molecular fingerprints are expected to occur, and a smooth curve was used to fit the chosen spectral points (Figure S12). This avoided overestimation of protein absorption and did not notably affect the value of ∆R^8^.

**Figure S12.** Extraction of ∆R from a measured spectrum.

**Supplementary references**

1 Lerner, M. B., Resczenski, J. M., Amin, A., Johnson, R. R., Goldsmith, J. I. & Johnson, A. T. C. Toward Quantifying the Electrostatic Transduction Mechanism in Carbon Nanotube Molecular Sensors. *J Am Chem Soc* **134**, 14318-14321 (2012).

2 Palik, E. D. & Ghosh, G. *Handbook of optical constants of solids*. (Academic Press, 1998).

3 Falkovsky, L. A. & Pershoguba, S. S. Optical far-infrared properties of a graphene monolayer and multilayer. *Phys Rev B* **76** (2007).

4 Yao, Y., Kats, M. A., Genevet, P., Yu, N. F., Song, Y., Kong, J. & Capasso, F. Broad Electrical Tuning of Graphene-Loaded Plasmonic Antennas. *Nano Lett* **13**, 1257-1264 (2013).

5 Lerner, M. B., Kybert, N., Mendoza, R., Villechenon, R., Lopez, M. A. B. & Johnson, A. T. C. Scalable, non-invasive glucose sensor based on boronic acid functionalized carbon nanotube transistors. *Appl Phys Lett* **102** (2013).

6 Kong, J. A. *Electromagnetic wave theory*. 2nd edn, (Wiley, 1990).

7 Azuelos, P., Girault, P., Lorrain, N., Poffo, L., Hardy, I., Guendouz, M. & Thual, M. Theoretical investigation of Vernier effect based sensors with hybrid porous silicon-polymer optical waveguides. *J Appl Phys* **121** (2017).

8 Yang, H. Y., Yang, S. N., Kong, J. L., Dong, A. C. & Yu, S. N. Obtaining information about protein secondary structures in aqueous solution using Fourier transform IR spectroscopy. *Nat Protoc* **10** (2015).
